# The Interaction between Chondroitin Sulfate and Dermatan Sulfate Tetrasaccharides and Pleiotrophin

**DOI:** 10.3390/ijms23063026

**Published:** 2022-03-11

**Authors:** María Jose García-Jiménez, Myriam Torres-Rico, José L. de Paz, Pedro M. Nieto

**Affiliations:** Glycosystems Laboratory, Instituto de Investigaciones Químicas (IIQ), cicCartuja, CSIC and Universidad de Sevilla, Americo Vespucio, 49, 41092 Sevilla, Spain; mariajose.garcia@iiq.csic.es (M.J.G.-J.); myriam.torres@iiq.csic.es (M.T.-R.); jlpaz@iiq.csic.es (J.L.d.P.)

**Keywords:** carbohydrate–protein interaction, pleiotrophin, chondroitin sulfate, GAG synthesis, transient NMR methods, STD-NMR spectroscopy

## Abstract

Pleiotrophin (PTN) is a neurotrophic factor that participates in the development of the embryonic central nervous system (CNS) and neural stem cell regulation by means of an interaction with sulfated glycosaminoglycans (GAGs). Chondroitin sulfate (CS) is the natural ligand in the CNS. We have previously studied the complexes between the tetrasaccharides used here and MK (Midkine) by ligand-observed NMR techniques. The present work describes the interactions between a tetrasaccharide library of synthetic models of CS-types and mimetics thereof with PTN using the same NMR transient techniques. We have concluded that: (1) global ligand structures do not change upon binding, (2) the introduction of lipophilic substituents in the structure of the ligand improves the strength of binding, (3) binding is weaker than for MK, (4) STD-NMR results are compatible with multiple binding modes, and (5) the replacement of GlcA for IdoA is not relevant for binding. Then we can conclude that the binding of CS derivatives to PTN and MK are similar and compatible with multiple binding modes of the same basic conformation.

## 1. Introduction

Pleiotrophin (PTN or HB-GAM) is a potent mitogenic cytokine [1] and, together with midkine (MK), constitutes the family of the neurite growth-promoting factors (NEGF) primarily involved in early neural growth and other physiological actions related to mitogenesis or inflammation [2]. They interact with extracellular glycosaminoglycans (GAG) that are fundamental for their activity through interactions with specific membrane receptors. This has been associated with, apart from neural regeneration, other biological events, including tissue repair, cancer metastasis, inflammation, bone development, or Alzheimer’s disease. PTN binds several membrane-specific receptors such as Receptor Protein Tyrosine Phosphatase (RPTP) β/ζ [3], syndecan-3, and anaplastic lymphoma kinase (ALK) [4]. This can be considered the habilitating step in signal internalization.

The structure of PTN has been elucidated by heteronuclear NMR [1,5,6] and is very similar to MK, consisting of two β-sheet domains connected by a flexible linker. Each β-sheet domain consists of three antiparallel β-strands stabilized by five disulfide bridges, with both containing a thrombospondin repeat I (TSR-I) motif. The central region consists of an intrinsically disordered region (IDR). It also has two lysine-rich clusters at the N- and C-terminal domains that appear as random flexible coils. TSR-I has been suggested to be responsible for PTN binding to heparin/heparan sulfate and N-sidecan [7] and to regulate PTN neurite outgrowth activity and synaptic plasticity as well as mitogenic and angiogenic activity (in particular the C-terminal TSR-I) [8].

**Figure 1:**
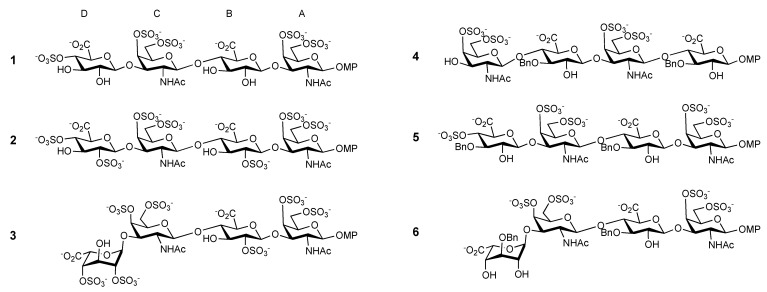
Tetrasaccharides studied in this work.

We have prepared six tetrasaccharides models of CS and hybrid sequences CS/DS (Chondroitin Sulfate/Desmatan Sulfate) carrying iduronate residues at the non-reducing end using chemical synthesis methods [9,10]. The first series is formed by CS type E, **1**, type T, **2**, and hybrid CS/DS, **3**, with an iduronate residue at the non-reducing terminal [9,10]. This series is followed by another with two benzyl moieties in position 3 of the Uronic acid ring, **4–6**. Their synthesis and 3D structures have been described previously (Figure 1) [9,10]. In this work, we describe the interaction between these tetrasaccharides and PTN using Fluorescence Polarization and NMR ligand-observed methods.

## 2. Results

We analyzed the relative binding affinities of **1**–**6** and PTN to calculate the IC_50_ values (half maximum inhibitory concentration) (Table 1). For this purpose, we employed our previously developed fluorescence polarization (FP) competition assay [9,10,11]. The results indicate that all the compounds studied have some affinity to PTN and that the presence of benzylic substituents increases affinity (compare **1** and **5**). In all cases, the affinity is less than to MK (see Table 1).

The three-dimensional structures of **1**–**6** have already been elucidated in a previous study of their interaction with midkine [10]. Here, we report on the NMR study of the interaction between tetrasaccharides **1**–**6** with PTN, using transfer NOE and STD-NMR. Tetrasaccharide **1** is a CS type E model, with sulfation in positions 4 and 6 of the galactosamine ring; **2** represents CS type T, with three sulfates at positions 2 of GlcA and 4 and 6 of GalN. Compound **3** is a DS / CS hybrid sequence with three rings with different characteristics (GlcA, IdoA, and GalNAc). Furthermore, it presents a sulphation pattern with the largest number of sulfate groups analog to **2**. The next three tetrasaccharides have two additional *O*-benzyl groups, previously found to enhance the affinity with midkine. Two of them, **4** and **5,** have the same sulfation pattern but an alternative sequence, and the third, **6**, has an iduronate at the non-reducing terminal, replacing the glucuronate. Due to synthetic strategies, **1**, **2, 3** and **5** have an additional sulfate group in position 4 of the non-reducing terminal. In all the cases, the reducing end is capped with a para-methoxyphenyl group to fix the beta stereochemistry at this terminal. The tetrasaccharides **4**–**6** should be considered mimetics of GAG, as they include two benzyl groups in position 3 of the uronic acids, and similar to **1**–**3,** they have been previously studied [9,10]. The preliminary values of IC_50_ confirm our strategy, as all of them show interaction with PTN (Table 1).

The three-dimensional structures of the tetrasaccharides have already been solved [9,10]. They have the same structural features as the CS that they model. All are helicoidal structures with high pitch and four residues per turn. They can also be considered linear structures, with the charged groups growing in opposite directions relative to the molecular axis. The introduction of benzyl substituents does not modify the 3D structures of the tetrasacharides [9,10].

**Figure 2:**
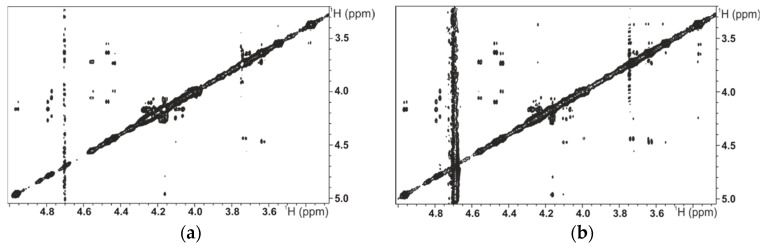
(**a**) NOESY of **1** (1.0 mM) at 200 ms mixing time, at 298 K in 250 μL PBS buffer (1×), (**b**) in the presence of 20 μM PTN (transfer NOESY). The vertical scale is the same.

We prepared samples for transient NMR experiments with a small amount of PTN in the presence of a significant excess of tetrasaccharides (molar ratio 1:20). First, we recorded the transfer-NOESY experiments at several mixing times to calculate the initial growth rate and estimate the ligand’s interprotonic distances into the complex [12]. We observed the same NOE pattern as in the free compounds; the distances of the interglycosidic NOEs were the same as those of the free ligand. In a previous work [12], we compared the NOE growing curves from the transfer NOESY sample with a similar model but without protein and showed the clear effect of the correlation time between both samples. As previously reported for MK [10], the ligands do not modify their 3D structures when they are complexed with PTN. See Supplementary Material and Figure 2.

**Figure 3:**
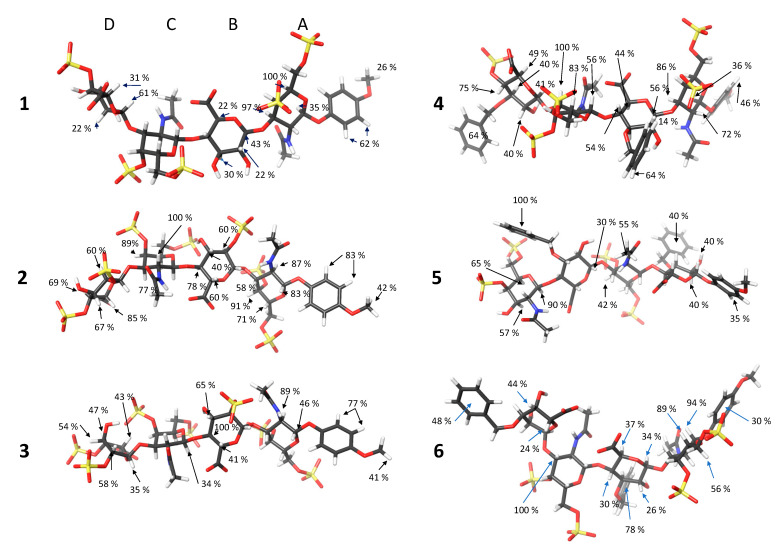
Relative STD_0_ values for **1**–**6** into randomly taken structures along time-averaged restrained molecular dynamics representative of the global disposition of substituents along the main molecular axis. The *p*-MethoxyPhenyl group is always oriented towards the right side.

We analyzed the results from the quantification of STD values. First, we calculated the initial growth rate to decouple differential relaxation from the transference of saturation. And then, to compare the values of all the tetrasaccharides, we use the relative STD values assigning 100% to the maximum STD found for the compound (Table 2). This methodology has been used for epitope mapping. Although, in principle, it can be safely used to compare potential binding modes, it should be undertaken with care, as it does not guarantee that the base peak (100%) varies from one compound to another or if it can be integrated (Figure 3).

The results are undefined compared with other systems involving carbohydrates and proteins interactions. There are two reasons for this low definition: the first is that it is due to alternative binding sites of the ligand into the protein, and second is that the orientation of the ligand into the same binding pocket on the complex is variable. Another potential reason could be the presence of multiple ligand shapes, but that is against the data obtained from transfer-NOE.

None of the PTN NMR structures deposited in the PDB database (2N6F) can form a complex similar to the one obtained with MK, where the tetrasaccharides were surrounded by the protein placed inside the pocket formed by the two folded domains. In another study of the interaction of a hexasaccharide using double-labeled PTN, the authors proposed an extended protein conformation, with the carbohydrate having contact with both protein domains [5]. However, the short length of the tetrasaccharides and the STD results are incompatible with a single face of the tetrasaccharide interacting with the protein in an extended conformation.

## 3. Discussion

We performed transient NMR experiments on the interaction between synthetic tetrasaccharides and PTN to explore the structure of the complex. They behave similarly to MK [9,10]. The transfer NOE is compatible with the same 3D structure free and bound: an extended chain, with *syn-Φ* glycosidic linkages that lead to a helicoidal, quasi-linear shape.

On the other hand, the STD results cannot be explained based on a single structure. They are compatible with an ensemble of structures without privileged orientation and not with a preferential binding site. The tetrasaccharides receive magnetization from all the spatial directions and ligand orientation. We propose two possible explanations for this behavior: the protein is folded surrounding the ligand, or the ligand jumps between binding sites in the link region or jumps between the two structured domains, NTD and CTD.

Then, PTN interacts with CS-E, CS-T, or hybrid DS/CS tetrasaccharides **1**–**6** and the mimetics thereof. In this paper, we have reported their PTN affinities, which are lower [9,10,11] than for MK but within the same range. The results described here link the affinity of PTN with CS to that of MK. In addition, we can also state that a minimum length of tetrasaccharides is needed for the interaction.

## 4. Materials and Methods

### 4.1. Fluorescence Polarization Assay

To calculate IC_50_ values, fluorescence polarization competition experiments were performed following our previously reported protocol [9,10,11]. Briefly, we recorded the fluorescence polarization from wells containing 20 µL of PTN (163 µM) and 10 µL of probe solution (fluorescein-labeled heparin-like hexasaccharide) in the presence of 10 µL of tetrasaccharide solutions with different concentrations. We used in these assays 384-well microplates from Corning. After shaking in the dark for 5 min, the fluorescence polarization was measured using a TRIAD multimode microplate reader (from Dynex), with excitation and emission wavelengths of 485 and 535 nm, respectively. The average polarization values of three replicates were plotted against the logarithm of tetrasaccharide concentration. The resulting curve was fitted to the equation for a one-site competition: y = A2 + (A1 − A2)/[1 + 10^(x − logIC_50_)^], where A1 and A2 are the maximal and minimal values of polarization, respectively, and IC_50_ is the tetrasaccharide concentration that results in 50% inhibition. At least two independent experiments were carried out for each IC_50_ calculation.

### 4.2. NMR

The NMR experiments were recorded in a BRUKER 600 AVANCE III HD spectrometer equipped with an ASCEND magnet and fitter with a QCI S3 H/F-C/N-D 05-Z cryoprobe, placed at the Plataforma de Interacciones Biomoleculares del cicCartuja (CSIC/US).

Free ligand assignment and structure elucidation have been performed and reported [9]. The structures of the tetrasaccharides complexed by PTN were elucidated based on ligand-observed methods, mainly transfer NOESY and STD. Standard pulse sequences from the Bruker library were used using phase-sensitive mode with gradient selection when possible. Samples were prepared for a 1:50 ratio protein-ligand (20 uM PTN, 1 mM ligand in 250 μL buffer PBS 1×). In Transfer NOESY experiments, zero quantum filter [13] and two modules of 180° pulses flanked with pulse field gradients to dephase unwanted magnetization were applied during mixing time [14]. STD experiments were acquired using Bruker standard library with a train of low power 50 ms length gaussian pulses spaced 1ms for saturation preceded by a purge module to eliminate residual magnetization from previous scans. This was followed by a short spin lock pulse to attenuate signals from the protein, and, when needed, solvent suppression using excitation sculpting was used [15]. All experiments were performed with alternate irradiation at 0.7 (on-resonance) and 40 ppm (off-resonance), further independent storage at 1.0, 1.5, 2.0, 3.0, 4.0, and 5.0 saturation times but with the same recycling time, and 16K point fid with 1024 accumulations were used.

## Figures and Tables

**Table 1:** IC_50_ values for the interaction between PTN and MK with synthetic tetrasaccharides **1**–**6** by FP.

Tetrasaccharide	PTN IC_50_ (μM)	MK ^a^ IC_50_ (μM)
**1**	338	136
**2**	24	10.6
**3**	12	8.0
**4**	81	31
**5**	45	27
**6**	25	n.d.

^a^ See Ref. [10].

**Table 2:** Relative and absolute STD_0_ values for **1**–**6**.

Atom	1 STD_0_ rel./abs.	2 STD_0_ rel./abs.	3 STD_0_ rel./abs.	4 STD_0_ rel./abs.	5 STD_0_ rel./abs.	6 STD_0_ rel./abs.
H1A	35/1.8	83/6.4	46/1.6	36/1.1		
H2A	87/6.8	87/6.8	89/3.1	72/2.1	40/1.1	56/1.0
H3A	97/4.9	58/4.6		86/2.6	40/1.1	89/1.6
H4A		91/7.1				
H5A	100/5.1	71/5.5				94/1.7
H1B	43/2.2			56/1.7		34/0.6
H2B	22/1.1	60/4.7		14/0.4	55/1.7	26/0.5
H3B	30/1.5	40/3.1	65/2.3			30/0.5
H4B		78/6.1	100/3.5	54/1.6	42/1.3	
H5B	22/1.1	60/4.7	41/1.4	44/1.3		37/0.7
H1C			34/1.2	56/1.7	30/0.9	
H2C		100/7.8				
H3C		77/6.0		83/2.5		100/1.8
H5C		89/7.0		100/3.0		
H1D	61/3.1		35/1.2	41/1.2	90/2.8	
H2D	22/1.1	60/4.7	58/2.0	40/1.2		
H3D	31/1.6	67/5.2	54/1.9	40/1.2	57/1.8	24/0.4
H4D		69/5.4	47/1.6	75/2.3		44/0.8
H5D		85/6.6	43/1.5	49/1.5	65/1.8	
CH_3_ (OMP)	26/1.3	42/3.3	41/1.4	31/0.9		20/0.4
Ph (OMP)	62/3.2	83/6.5	77/2.7	46/1.4	35/1.1	30/0.6
CH_2_Ph				64/1.9	**100/3.1**	78/1.4
CH_2_Ph				64/1.9	40/1.2	48/0.9

## Data Availability

Not applicable.

## Supplementary Materials

The following supporting information can be downloaded at: https://www.mdpi.com/article/10.3390/ijms23063026/s1.
