# Supplementary material for: The Interaction between Chondroitin Sulfate and Dermatan Sulfate Tetrasaccharides and Pleiotrophin
Source: Int J Mol Sci. 2022 Mar 11;23(6):3026. doi: 10.3390/ijms23063026 (PMC8955691; doi:10.3390/ijms23063026)
Supplement: Supplementary file 1 [file ijms-23-03026-s001.zip › ijms-1622432-supplementary.pdf]

# The interaction between CS and DS/CS tetrasaccharides and Pleiotrophin

M. Jose García-Jiménez,[1] Myriam Torres-Rico,[1] José L. de Paz [1] and Pedro M. Nieto [1 *]

# Fluorescence Polarization assays

**Figure S1: (compound 1).** Three independent competition experiments were performed. The obtained IC50 values, the average and the standard deviation from these experiments are given. As an example, a representative competition curve is also shown. The same for Figures S2-S6.

| | |
|---|---|
| IC50 (first measure) | 338 |
| IC50 (second measure) | 346 |
| IC50 (third measure) | 331 |
| Average | 338 |
| Standard deviation (std) | 7.5 |

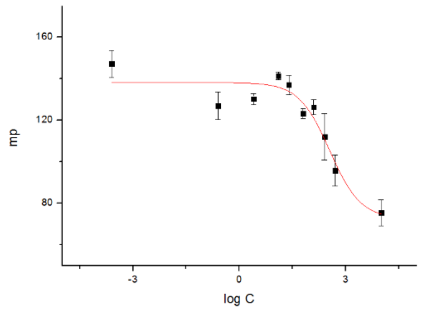


**Figure S2: (compound 2)**

| | |
|---|---|
| IC50 | 27 |
| IC50 | 28 |
| IC50 | 16 |
| Average | 24 |
| std | 7 |

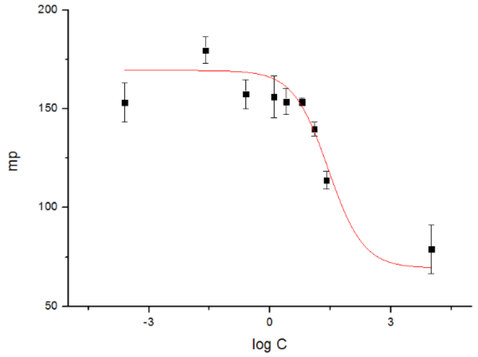

**Figure S3: (compound 3)**

| | |
|---|---|
| IC50 | 10 |
| IC50 | 16 |
| IC50 | 11 |
| Average | 12 |
| std | 3 |

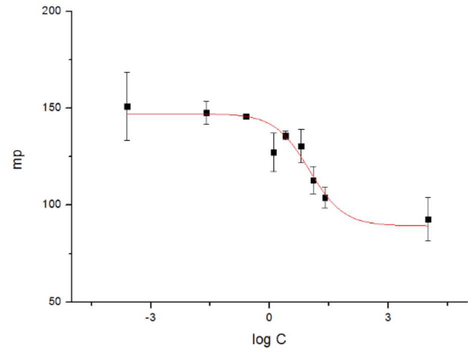


**Figure S4: (compound 4)**

| | |
|---|---|
| IC50 | 83 |
| IC50 | 78 |
| Average | 80.5 |
| std | 3.5 |

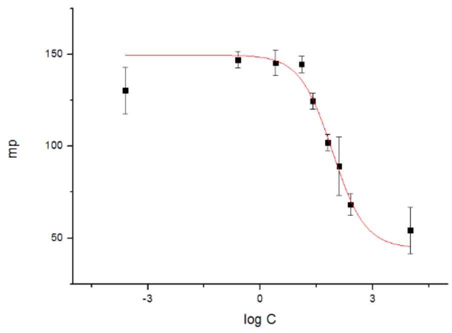

**Figure S5: (compound 5)**

| | |
|---|---|
| IC50 | 39 |
| IC50 | 48 |
| IC50 | 50 |
| Average | 45 |
| std | 6 |

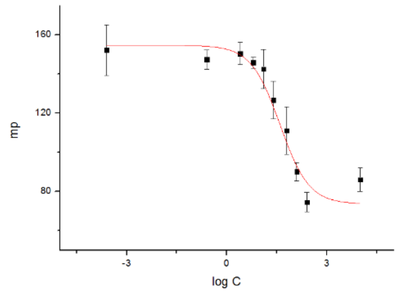


**Figure S6: (compound 6)**

| | |
|---|---|
| IC50 | 20 |
| IC50 | 12 |
| IC50 | 44 |
| Average | 25 |
| std | 17 |

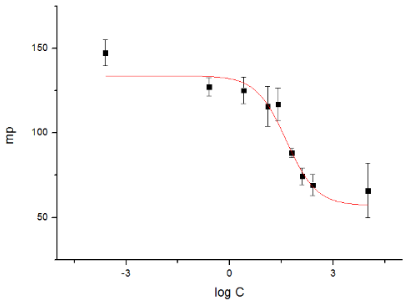

# Transfer NOESY, mixing time = 200ms

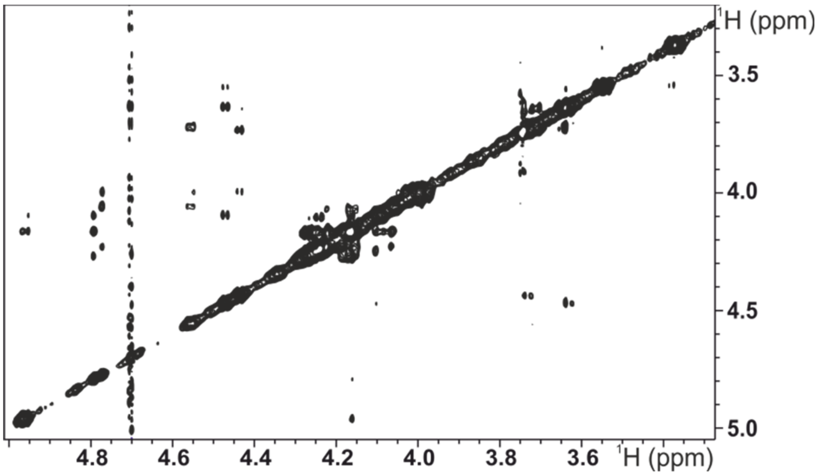


**Figure S7.** Transfer NOESY experiment of **1** mixing time 200ms. For experimental conditions see Materials and Methods section.

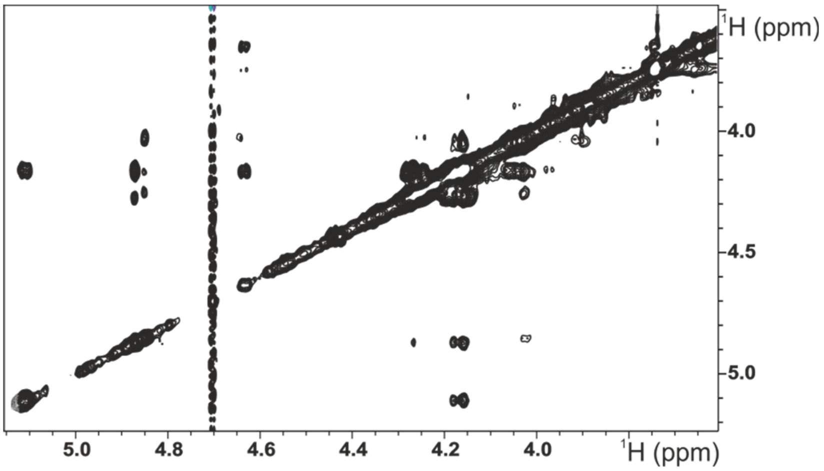


**Figure S8.** Transfer NOESY experiment of **2** mixing time 200ms. For experimental conditions see Materials and Methods section.

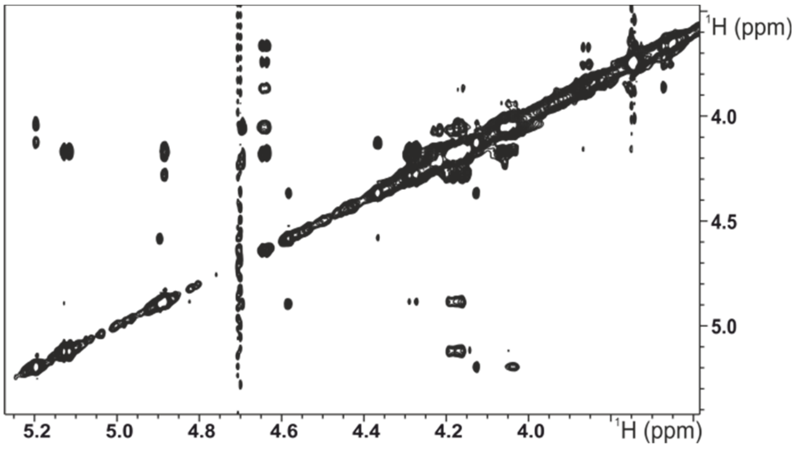


**Figure S9.** Transfer NOESY experiment of **3** mixing time 200ms. For experimental conditions see Materials and Methods section.

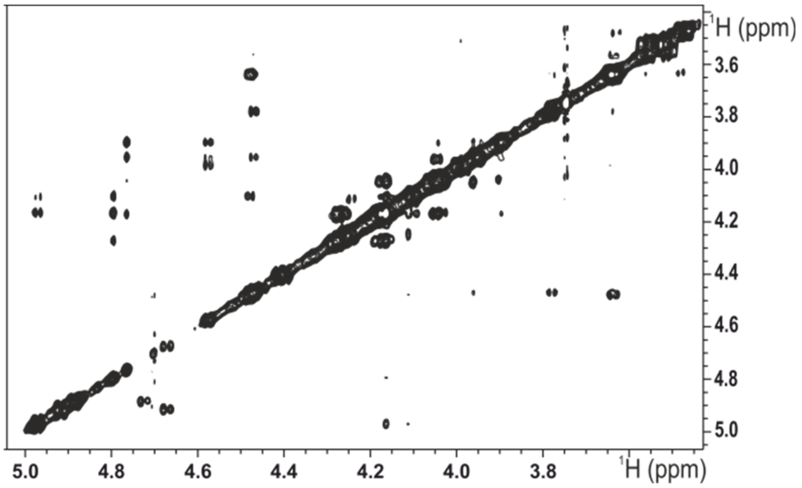


**Figure S10.** Transfer NOESY experiment of **4** mixing time 200ms. For experimental conditions see Materials and Methods section.

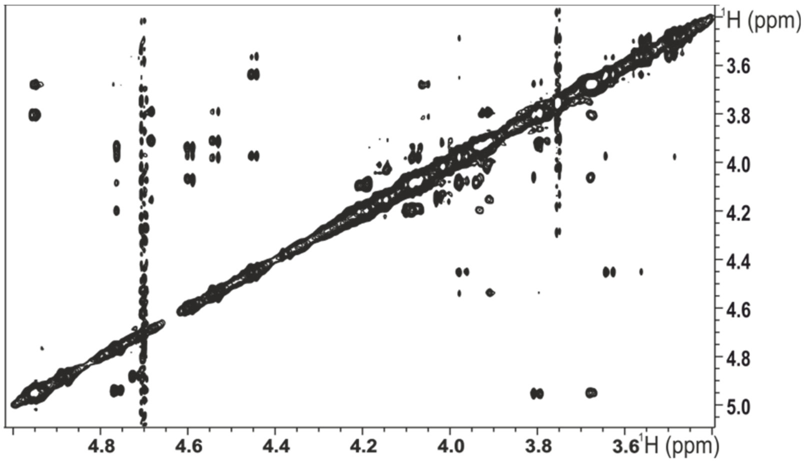


**Figure S11.** Transfer NOESY experiment of **5** mixing time 200ms. For experimental conditions see Materials and Methods section.

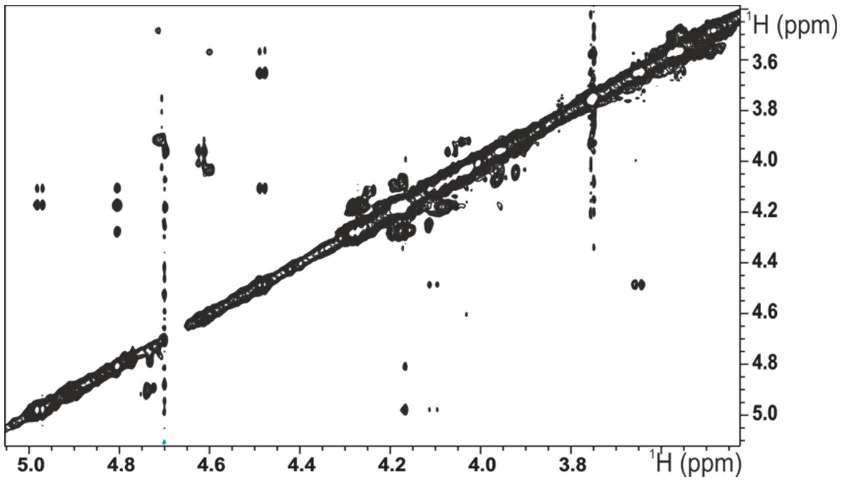


**Figure S12.** Transfer NOESY experiment of **6** mixing time 200ms. For experimental conditions see Materials and Methods section.

## STD-NMR

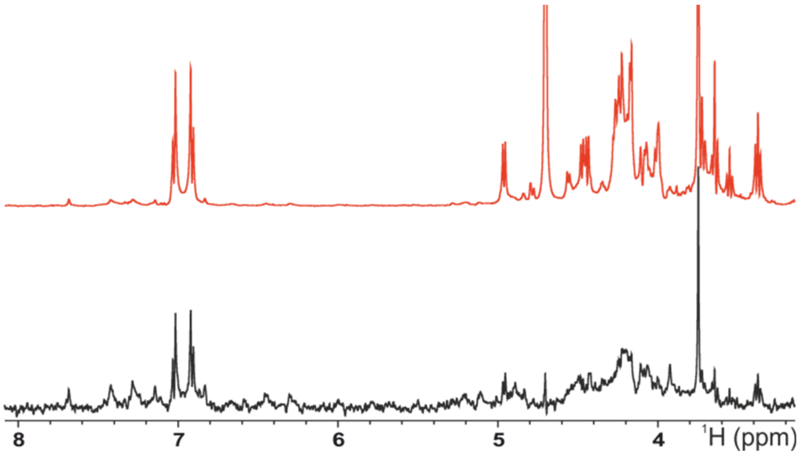


**Figure S13.** STD-NMR experiment of **1** (red) reference $^{1}H$ (black), irradiation time 4s, experimental conditions see Materials and Methods section.

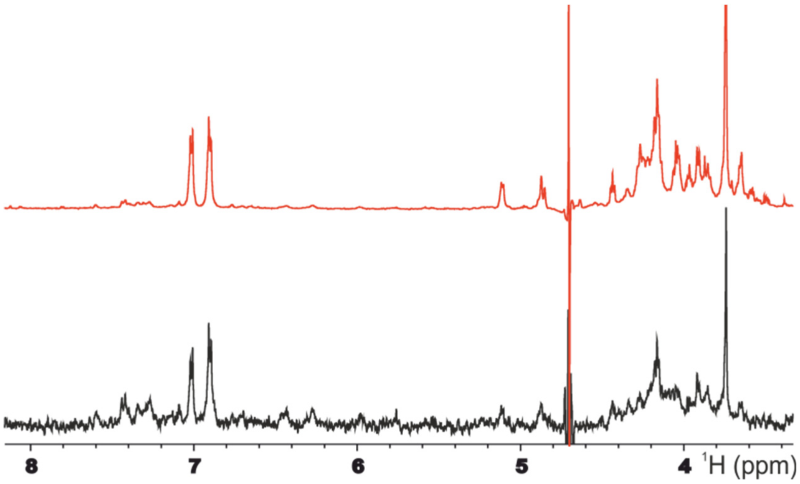


**Figure S14.** STD-NMR experiment of **2** (red) reference $^{1}H$ (black), irradiation time 4s, experimental conditions see Materials and Methods section.

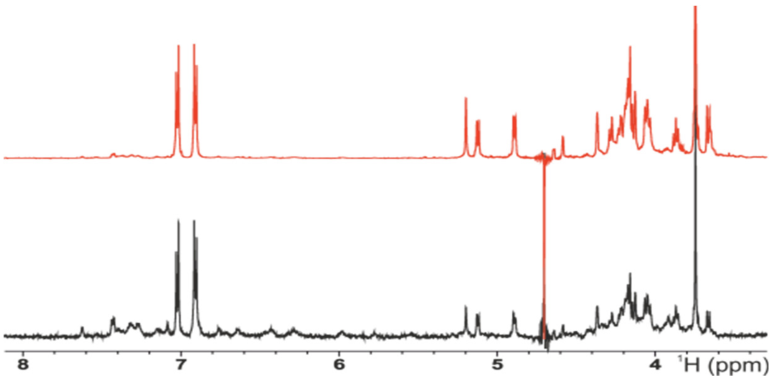


**Figure S15.** STD-NMR experiment of **3** (red) reference $^{1}$H (black), irradiation time 4s, experimental conditions see Materials and Methods section.

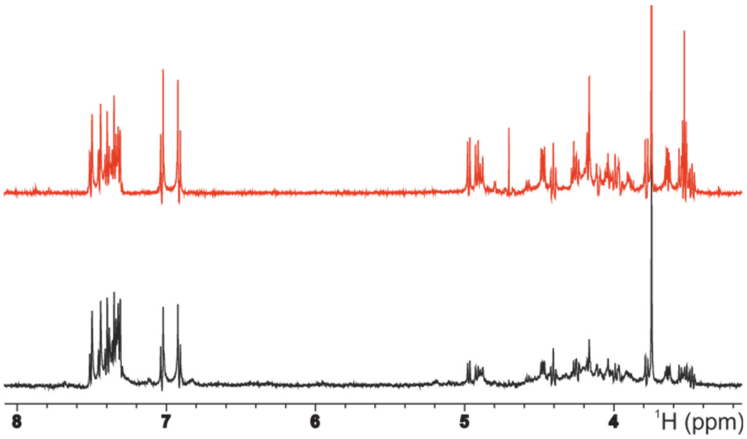


**Figure S16.** STD-NMR experiment of **4** (red) reference $^{1}$H (black), irradiation time 4s, experimental conditions see Materials and Methods section.

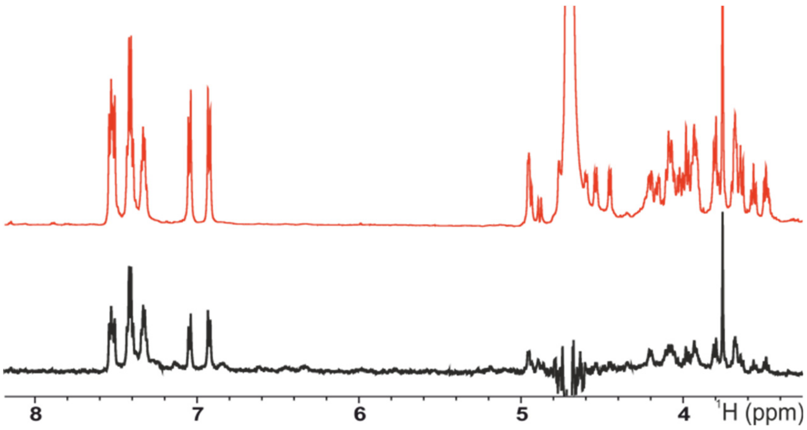


**Figure S17.** STD-NMR experiment of **5** (red) reference $^{1}H$ (black), irradiation time 4s, experimental conditions see Materials and Methods section.

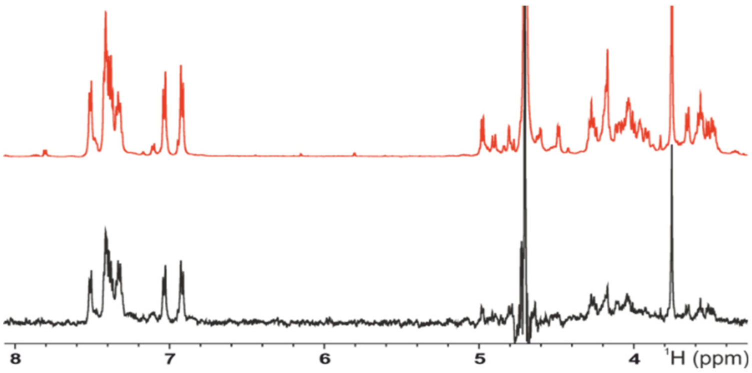


**Figure S18.** STD-NMR experiment of **6** (red) reference $^{1}H$ (black), irradiation time 4s, experimental conditions see Materials and Methods section.

# STD-NMR growth rates

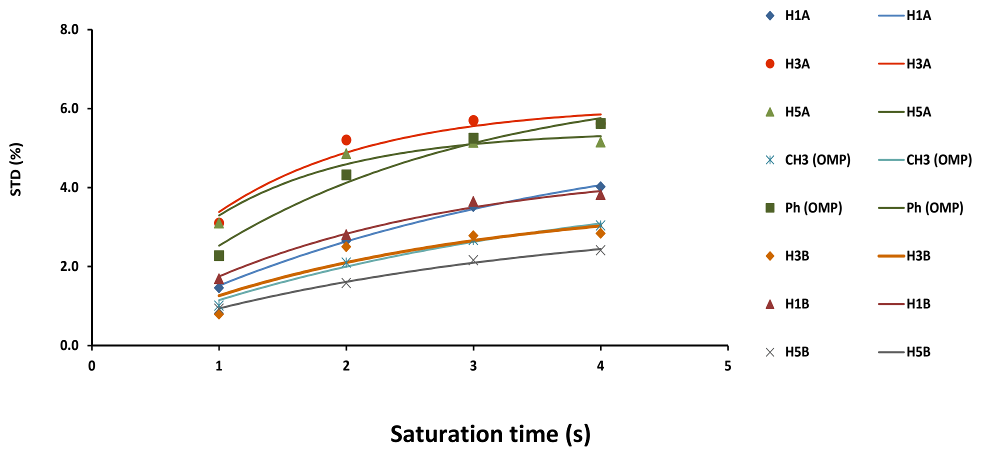


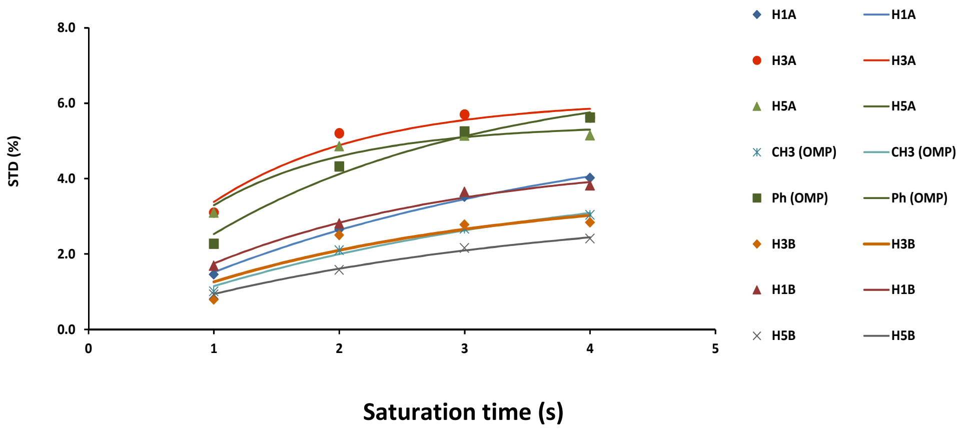


**Figure S19.** STD-NMR growth rates of **1**. Experimental STD values are indicated by the corresponding symbol. The value of the initial growth rate was obtained by the ajustment of the experimental values to the exponential equation Box Lucas.

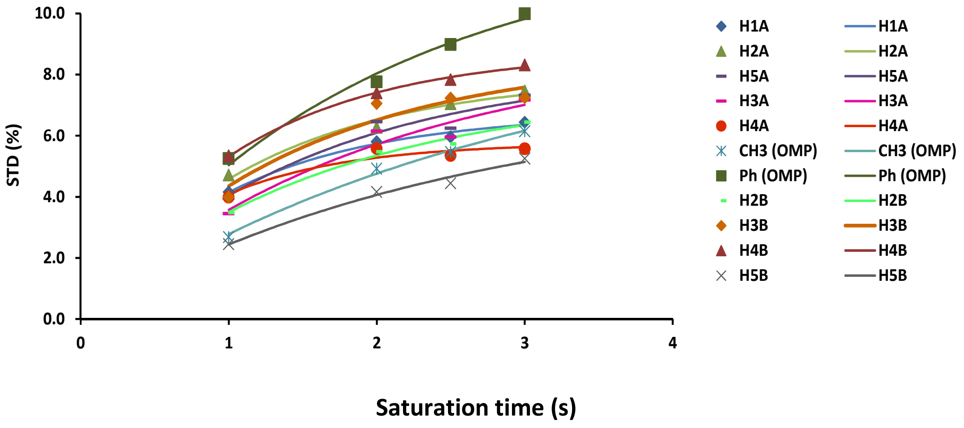


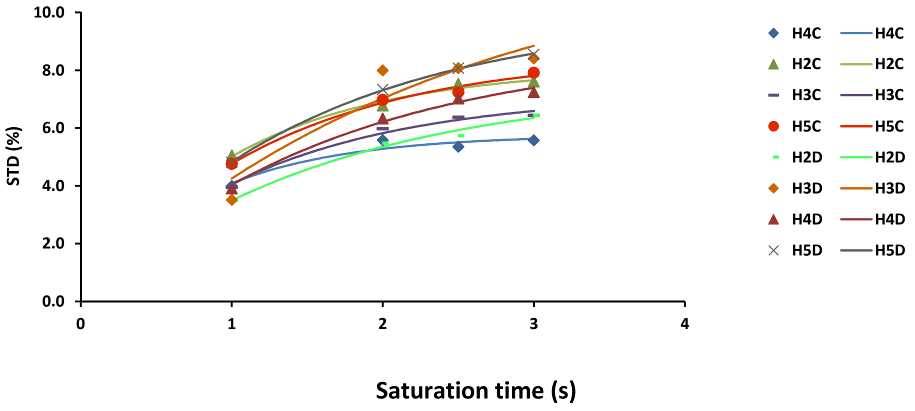


**Figure S20.** STD-NMR growth rates of **2**. Experimental STD values are indicated by the corresponding symbol. The value of the initial growth rate was obtained by the ajustment of the experimental values to the exponential equation Box Lucas.

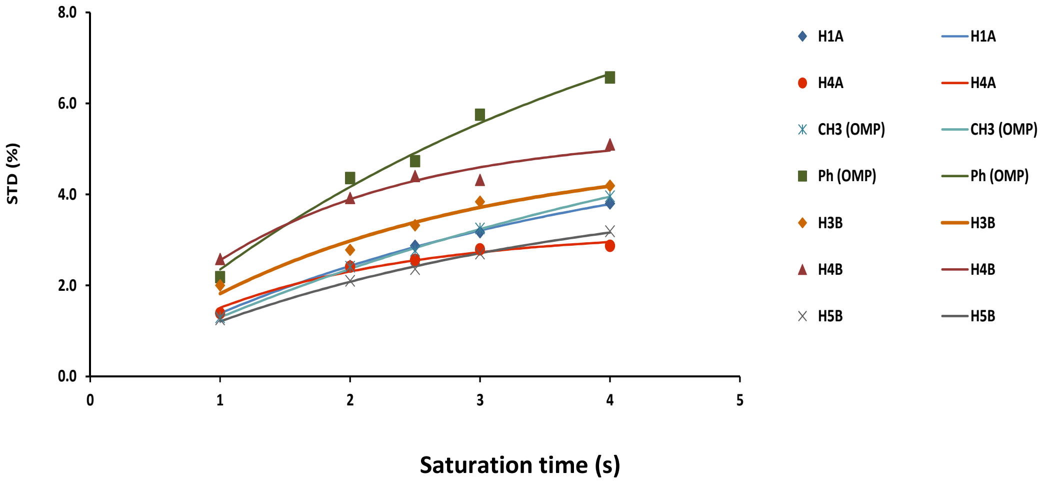


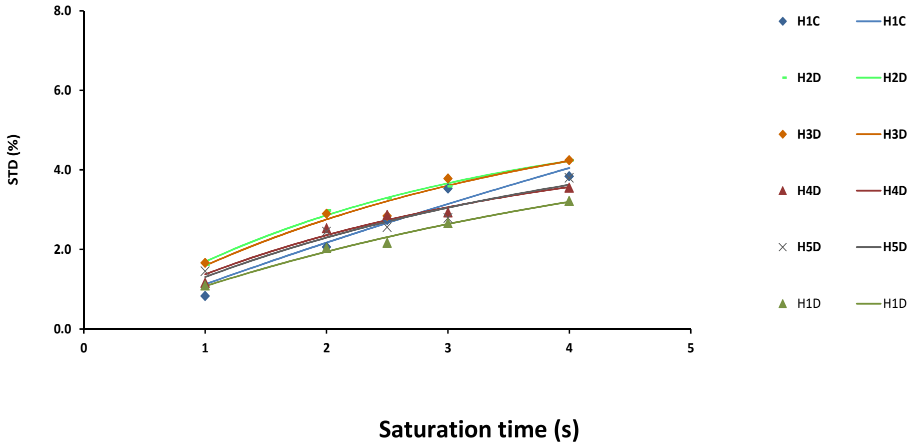


**Figure S21.** STD-NMR growth rates of **3**. Experimental STD values are indicated by the corresponding symbol. The value of the initial growth rate was obtained by the ajustment of the experimental values to the exponential equation Box Lucas.

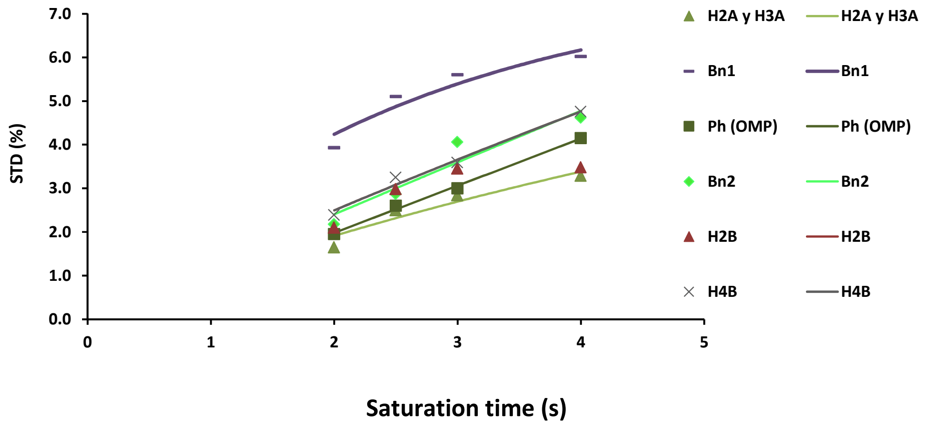


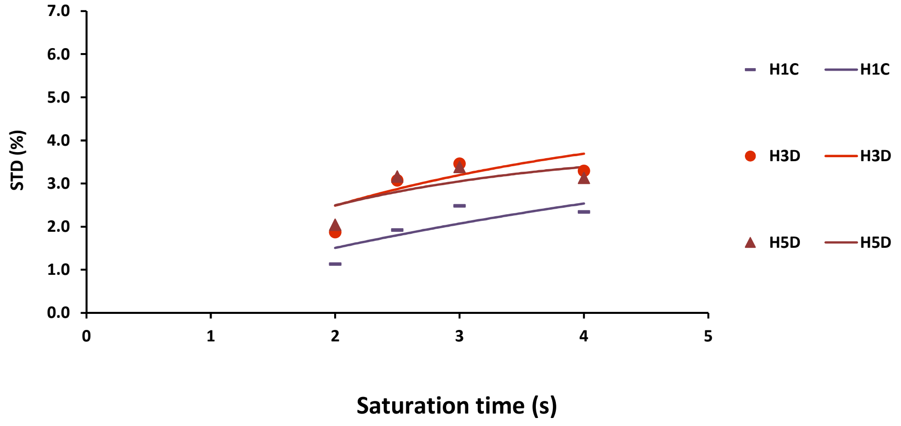


**Figure S22.** STD-NMR growth rates of **4**. Experimental STD values are indicated by the corresponding symbol. The value of the initial growth rate was obtained by the ajustment of the experimental values to the exponential equation Box Lucas.

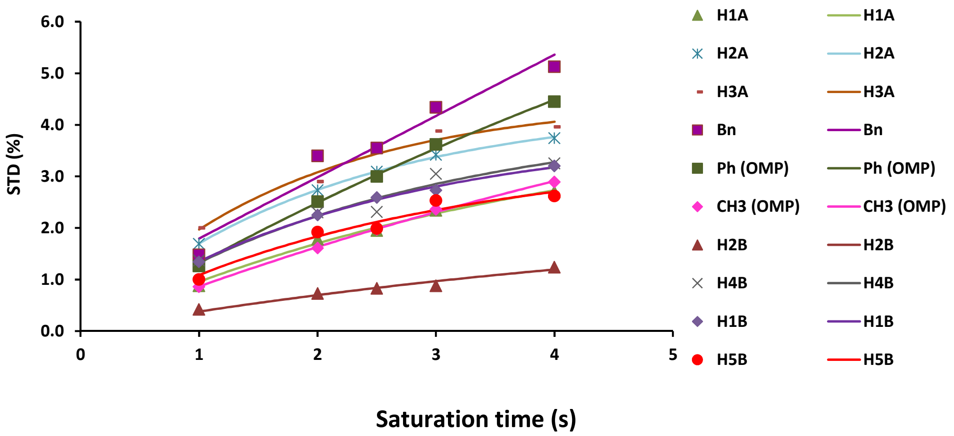


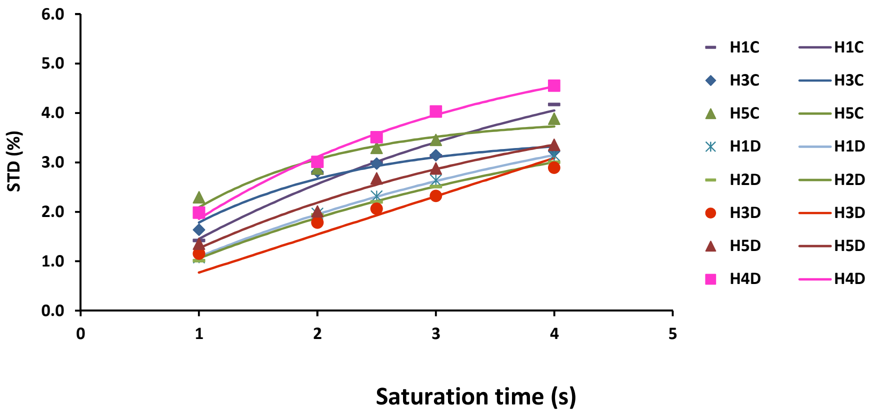


**Figure S23.** STD-NMR growth rates of **5**. Experimental STD values are indicated by the corresponding symbol. The value of the initial growth rate was obtained by the ajustment of the experimental values to the exponential equation Box Lucas.

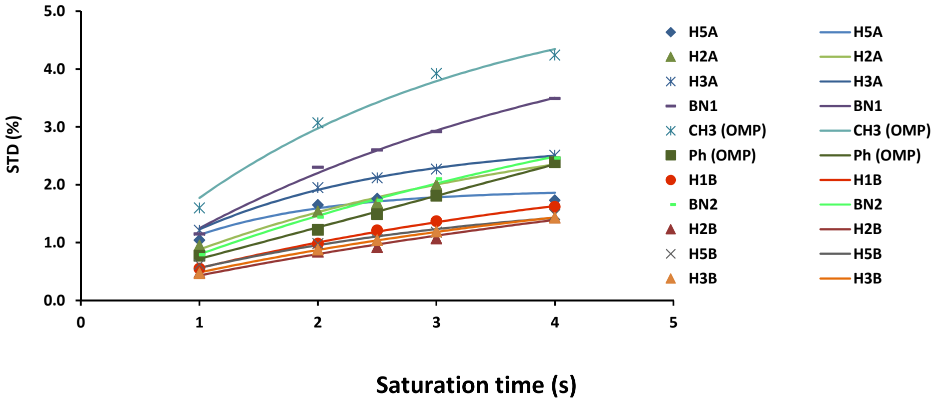


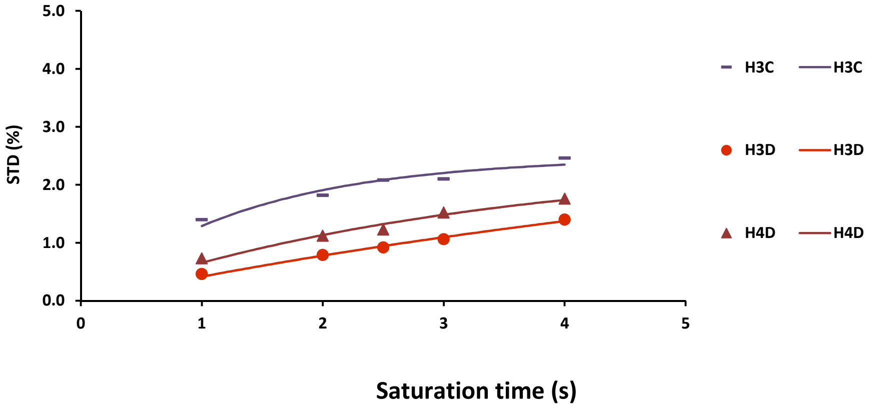


**Figure S24.** STD-NMR growth rates of **6**. Experimental STD values are indicated by the corresponding symbol. The value of the initial growth rate was obtained by the ajustment of the experimental values to the exponential equation Box Lucas.
